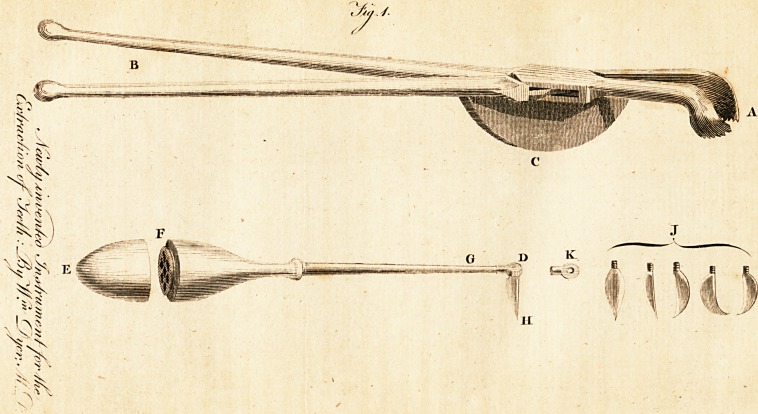# Remarks on the Extraction of Teeth, with a View to Lessen the Danger of That Operation; and a Description of a New Instrument for Drawing Teeth; Including the Account of an Improved Scarificator

**Published:** 1799-03

**Authors:** W. Dyer

**Affiliations:** Aberdeen


### *Remarks on the Extraction of Teeth, with a View to lessen the Danger of that Operation; and a Description of a new Instrument for Drawing Teeth; including the Account of an improved Scarificator:—Communicated by* Dr. W. Dyer, *of Aberdeen.*

[ *With a Plate.* ]

EVERY perfon converfant in the operations of furgery, knows how to extract teeth; but few, comparatively, who perform this operation, confider the danger that attends it. If I wifh to draw a nail, (which having been retained for fome time in a wall, and, by means of the oxygen contained in the atmofphere, converted partly into an oxyde, nearly refembles a carious tooth) I can extract it with the affiftance of a common hammer, by fixing the nail between the claw; (this is eafily done, as the latter adapts itfelf to any fize, being nearly of the form of the letter V) then forcing the handle to one fide, I thereby form a fulcrum on the edge of the hammer, or the fide of the claw, and by the power which the long handle or lever affords, I am enabled either to extract the nail, or perhaps to break it; but fhould there be the leaft appearance of fo doing, I then immediately force the handle to the other fide, and on taking another hold, the nail, in all probability, will come out complete or entire. Such is precifely the cafe with regard to the drawing of teeth; for whenever the pull is too great to venture upon, by changing the claw of the inftrument, and fixing it on the other fide, the tooth being already loofened by the firft attempt, a fmall degree of force only will be requifite to bring it to the other fide, and it will then come out along with the inftrument; yet never without fome injury being done to the alveolar procefs. To be convinced that this muft be the cafe, we need only confider the pofition of the hammer in pulling the nail, as it is more completely expofed to view, both inftruments being perfectly fimilar in their application and principles. On the firft attempt to pull, we find that the nail begins to bend to one fide; an additional force brings it out a little, but in what direction?—not in a ftraight line, but a curved one, forming a fegment of the circle, which would be defcribed by placing one leg of a compafs at the fulcrum, or that part where the hammer refts, and extending the other leg to the nail; then drawing a circle by means of this known radius, the nail when extracted, would exactly correfpond with a part of the circumference of the circle drawn. But as the human teeth do not bend, being different in their texture from that of the metal of which the nail is compofed; and as the bed or focket in which they are lodged, is likewife different from the wood in which the nail is inclofed, it is not to be wondered at, that one or other of them will give way, confidering the fhort turn which the inftrument muft, from its conftruction, defcribe.

Le

Let us suppose the claw placed (either outside or inside) upon the tooth, with the point of it as near to the gum as possible, the rest or fulcrum also being placed on the opposite side, as near to the jaw as may be without resting upon it, then by taking hold of the handle of the instrument in the right hand, we give it a twist, which we shall suppose brings it out, yet so that one or other of the circumstances already mentioned will take place, viz. the tooth broke and part of the fang retained in the cavity, and if so, the former complaints very often will continue, and sometimes increase beyond endurance, or if the tooth be whole, a considerable splinter of the alveolar process will be brought out along with it.

These inconveniences, not to mention the great pain and dangerous consequences that frequently ensue, have not passed unnoticed by professional men, in every country where the key instrument has been in use—an instrument too well known to require a description here, as no person capable of judging of its imperfections can be ignorant of its construction. The instrument used a century ago for the same purpose does not materially differ from the one at present in use; it no doubt has undergone a variety of forms, but the principle remains the same. Among the numerous attempts to improve it, the only one, not materially different from the old instrument, and which merits particular notice, is that proposed by Mr. SAVIGNY, surgical instrument maker, in London, and described in the 7th volume of "*Medical Facts and Observations.*" That gentleman has certainly heard many complaints, and is himself well qualified to judge of the imperfections of the old instrument, but the improvement which he wishes to introduce, (consisting of a small cylinder or bolster on the end of the instrument, placed on the tooth as near to the process as possible, the claw being fixed to the circumference of this cylinder) by no means performs what he intends or says, viz. "the extraction of the tooth in nearly a perpendicular direction".

A trial of the instrument is scarcely necessary to prove this; a simple inspection of the plate may suffice to convince us that the end proposed is not here attained, and also that his instrument is not materially different from the common one, either in its direction, or power of action. In a late publication (the "*Philosophical Magazine*") I read with great satisfaction the announcement of a description and plate of a new German key for extracting teeth, in hopes that it would effectually supersede any attempt of mine towards improvement, or at least, that I should receive from it such additional information as would enable me perhaps to improve my own; but now, after having examined the plate and read the description, I am concerned to find that not one improvement is even attempted. An instrument exactly similar to the one here mentioned, I have not only seen many years ago used ufed by others, but I have alfo actually ufed myfelf—perhaps it may be new to its recommender; it can fcarcely be fo, however, to an experienced medical practitioner. He fays, "the improvements introduced into this inftrument are fuch as, I hope, will be found to remedy the defects complained of in all former ones; and the facility with which a tooth can be drawn by its means, will, I am fure, render its adoption in practice univerfal, as foon as it fhall be generally known." In oppofition to which I am forry to obferve, that the imperfections of this inftrument have been too long known and felt, not to enable us to decide whether it correfpond in efficient practice to the encomiums he is pleafed to beftow on it. It is fcarcely neceffary to mention, that the only advantage which this inftrnment poffeffes over thofe in common ufe, is the fhort time that is required in changing the claw from one fide of the tooth to the other; but this is not always neceffary, and it is only an additional expence to the price of the inftrument, without producing any advantage equivalent thereto. The principal objection to it, and the difagreeable confequences that enfue from the ufe of all other inftruments, which have been contrived for extracting teeth, is certainly the following; the fhort lateral turn or curve which the inftrument defcribes when in action:—But how is this to be remedied? We can eafily find out imperfections, and yet the means hitherto employed for the removal of them have not been attended with that fuccefs, which the importance of the object deferved. All who have confidered the common key inftrument, or indeed any one with which we have been yet made acquainted, will agree, that the fudden turn, defcribed by the tooth or inftrument while extracting, occafions moft of the inconveniences attending the operation: when this is known, the practitioner will naturally fay to himfelf, the main thing required is to pull the tooth in a perpendicular direction, or rather in the direction of its axis; but when again we come to confider how this is to be done, a queftion occurs, what inftrument can be ufed, which will have fufficient power, and yet be applicable in fo confined a fituation? The folution of this feems infurmountable.

Several ingenious men have attempted to mafter the difficulty, but none, as I know, have been fuccefsful, and although I am inclined to think that, what I have to propofe will anfwer the intended purpofe, perhaps I may be deceived; and if I fhould, it affords me fome confolation to reflect that in this refpect I am not fingular: and I fincerely wifh and hope that the hint may be, by fome perfon of ingenuity, applied with more advantage and neatnefs to the purpofe intended.

We have already confidered the inconveniences attending the ufe of the key inftrument as commonly applied, by its fudden turning the tooth to one fide fide; what we have now to confider is, whether it be poffible to extract it in the direction of its axis, or in a perpendicular direction, and in what way? If we again confider how the hammer is employed in extracting a nail in another direction, we fhall at once conclude, that another inftrument, or at leaft one conftructed on different principles, will be requifite for that purpofe. If I wifh to pull the nail in a more perpendicular direction, fix it firft in the claw, then by caufing the end or folid part of the hammer to reft upon the wall, I do not force it to one fide, but pull exactly in the direction of the claw, by which means the nail is raifed nearly in a perpendicular direction, or rather in the direction of a fegment of the circle formed by the point of the claw of the hammer where the nail refts, taking the folid part or other end of it, where the fulcrum is, for the centre. In nearly a fimilar way, would I propofe teeth to be extracted; the inftrument, however, for this purpofe will be beft underftood from the annexed drawing.

A B reprefents the inftrument nearly of its proper fize, and refembles in a great meafure the common ftump forceps; attached to the forceps at C is a femi-circular piece of wood or metal, the under part of which is ftuffed and covered with leather: this femi-circular piece is fo conftructed that it may be flipped off at pleafure, and a larger or fmaller one adapted, as the cafe may require; whenever this inftrument is to be ufed, it is requifite, in the firft place, to obferve that there be no vacancy between the tooth to be extracted and the front teeth; then, having previoufly well feparated the gum from the tooth, the point of the inftrument is to be applied on each fide of the tooth as low down, and taking as firm a hold of it, as poffible; then by depreffing the handle, at the fame time taking care not to lofe hold of the tooth, the femi-circular piece refts upon the anterior teeth, and forms a fulcrum, yet it produces a very different effect at the point of the inftrument, which a common prop would do; for by means of it, the tooth when raifed, defcribes, not the circle which would be formed by taking the diftance between the point of the inftrument and the fulcrum, but one that is of a much larger radius; which of courfe comes nearer the direction wanted, the perpendicular. I am well aware, that one great objection will be offered to the ufe of this inftrument, and that is, in cafes where one, two, or three teeth are wanting, and where there is nothing to reft upon but the gum; even in fuch cafes, this inconvenience may be eafily obviated by having a flat piece of metal with a fmall handle attached to the fide; this flat piece being ftuffed in the under fide and covered with leather, fhould be placed upon the gum; then the femi-circle of the inftrument refts upon it as upon the teeth, and in this way the inftrument may be ufed with as much facility in the one cafe as in the other.

The

The force neceſſary to draw a tooth is not ſo great as one would be apt to imagine, provided the cords (if I may be allowed ſo to call the gum) be completely ſeparated from the tooth, which by the bye is ſeldom done by any operators.—Indeed, moſt perſons that are in the practice of extracting teeth, complain of the difficulty of dividing the gum from the tooth completely; and a very eminent writer on this ſubject ſays, "It is a common practice to divide the gum from the tooth before it is drawn, which is attended with very little advantage, becauſe at beſt it can only be *imperfectly done*;" and he adds, "But if ſuch a ſeparation, as can be made, ſaves any pain in the whole of the operation, I ſhould certainly recommend it, and at leaſt in ſome caſes, it might prevent the gum from being torn."—Now what I wiſh to advance on this ſubject is to endeavour to point out a method of performing this part of the operation *completely*; for the author above quoted certainly does not mean to affirm that it is uſeleſs, but only that the method at preſent in uſe does not anſwer the intention. If we examine any of the ſcarificators commonly uſed for the purpoſe, we ſhall ſoon be convinced, that they cannot anſwer the end fully, and we ſhall find alſo that they are ſo conſtructed as not to admit of being applied exactly round the tooth: for, with all the care which we can poſſibly exert to go round the convexity of the tooth, ſtill there will be ſome part which cannot be come at. But, by ſubſtituting the following ſimple ſcarificator, I hope all theſe defects will be completely remedied. D. E. Fig. 2. repreſents the inſtrument I propoſe, of its proper ſize; the handle at F. unſcrews, and in it is contained three, four, or ſix blades, or ſcarificators, any one of which may be applied at pleaſure; for by unſcrewing the ſtem at G. out of the knob K. the blade H. alſo unſcrews, and another of a different form may be ſubſtituted, and can be made *dead* faſt in any direction by means of the ſcrew at G. ſo that it may be exactly adapted to the curvation of the tooth, and of courſe the gum ſeparated more completely than by any former ſcarificator. I. repreſents blades of different forms; one form being found, on ſome occaſions, more convenient than another, and as any of them will fit the ſcrew at D. they may be made faſt in any direction, by means of the ſcrew in the point of the ſtem at G.—K. repreſents the ſmall button or end-piece unſcrewed from the ſtem, and without a blade.

*Aberdeen*, W. DYER.

*Jan.* 25*th*, 1799.

* Mr. JOHN HUNTER "*On the Diseases of the Teeth,*" p. 90.

## Figures and Tables

**Fig. 1. f1:**